# pH responsive N-succinyl chitosan/Poly (acrylamide-co-acrylic acid) hydrogels and *in vitro* release of 5-fluorouracil

**DOI:** 10.1371/journal.pone.0179250

**Published:** 2017-07-05

**Authors:** Shahid Bashir, Yin Yin Teo, Sumaira Naeem, S. Ramesh, K. Ramesh

**Affiliations:** 1 Department of Chemistry, Faculty of Science, University of Malaya, Kuala Lumpur, Malaysia; 2 Centre of Ionics University of Malaya, Department of Physics, Faculty of Science, University of Malaya, Kuala Lumpur, Malaysia; Kyoto Daigaku, JAPAN

## Abstract

There has been significant progress in the last few decades in addressing the biomedical applications of polymer hydrogels. Particularly, stimuli responsive hydrogels have been inspected as elegant drug delivery systems capable to deliver at the appropriate site of action within the specific time. The present work describes the synthesis of pH responsive semi-interpenetrating network (semi-IPN) hydrogels of N-succinyl-chitosan (NSC) via Schiff base mechanism using glutaraldehyde as a crosslinking agent and Poly (acrylamide-co-acrylic acid)(Poly (AAm-co-AA)) was embedded within the N-succinyl chitosan network. The physico-chemical interactions were characterized by Fourier transform infrared (FTIR), X-ray diffraction (XRD), thermogravimetric analysis (TGA), and field emission scanning electron microscope (FESEM). The synthesized hydrogels constitute porous structure. The swelling ability was analyzed in physiological mediums of pH 7.4 and pH 1.2 at 37°C. Swelling properties of formulations with various amounts of NSC/ Poly (AAm-co-AA) and crosslinking agent at pH 7.4 and pH 1.2 were investigated. Hydrogels showed higher swelling ratios at pH 7.4 while lower at pH 1.2. Swelling kinetics and diffusion parameters were also determined. Drug loading, encapsulation efficiency, and in vitro release of 5-fluorouracil (5-FU) from the synthesized hydrogels were observed. In vitro release profile revealed the significant influence of pH, amount of NSC, Poly (AAm-co-AA), and crosslinking agent on the release of 5-FU. Accordingly, rapid and large release of drug was observed at pH 7.4 than at pH 1.2. The maximum encapsulation efficiency and release of 5-FU from SP2 were found to be 72.45% and 85.99%, respectively. Kinetics of drug release suggested controlled release mechanism of 5-FU is according to trend of non-Fickian. From the above results, it can be concluded that the synthesized hydrogels have capability to adapt their potential exploitation as targeted oral drug delivery carriers.

## Introduction

Hydrogels are three dimensional crosslinked polymer networks. Classification of hydrogels depends upon source of materials used during synthesis, method of synthesis and crosslinking category [1]. The amount of water absorbed by the hydrogels in their swollen state depends upon the materials used during synthesis and also the behavior of crosslinking agent [2, 3]. Water absorption is the main property of hydrogels which briefly explains its usage [4]. The reason is that high water absorption efficiency has promoted hydrogel to imitate the biological tissues with high biocompatibility. This nature reduces the inflammatory reaction in biological tissues [5, 6].

Controlled drug delivery system deliver drug in the body deliberately at preordained rates within a calculated period of time [7]. It is a worthwhile if the drug is administered into the body and matches accurately the physiological requirements. In drug release systems, oral route is significantly comfortable and suitable approach for administration of drugs [8]. The pH sensitivity plays a major role in oral drug delivery as a result of different pH in the body segments. The hydrogels possess the characteristics of pH sensitivity [9].

Basically, pH sensitivity and oral delivery of drugs from hydrogels depend upon the composition of hydrogels. A diverse range of polymer compositions have been used to fabricate hydrogels. The compositions can be divided into natural, synthetic or combination of two classes [10]. Natural polymers are biocompatible, non-toxic and excellent carriers of drugs, in addition to biodegradability which is the inherent advantage of natural polymers [11]. However, natural polymer hydrogels have very weak mechanical strength. Among natural polymers, chitosan is a semi-synthetic natural polysaccharide attained from deacetylation of chitin under alkaline conditions [12–14]. Chitosan exhibits outstanding properties for instance biodegradability, biocompatibility, non-toxicity, mucoadhesivity, and non-carcinogenicity. The non-toxic degradable products of chitosan also play a significant role in extensive exploitation of biomedical formulations [15, 16]. Unfortunately, the uncontrollable biodegradability, less pH-sensitivity, release of drug in the stomach, and solubility in acidic medium prevented its momentous development in drug delivery. Therefore, chemical modification of chitosan seems to be an encouraging prospect in order to improve the drawbacks of chitosan [17]. Therefore, chitosan derivatives have significantly increased the scientific interest due to their outstanding properties [18–20]. N-succinyl chitosan (NSC) is an acyl derivative of chitosan that is a biocompatible, biodegradable, and highly pH sensitive [21]. Furthermore, the negatively charged carboxylate ions in N-succinyl chitosan promote the mucoadhesive properties. Due to these properties, NSC received striking attention in drug delivery applications [22]. NSC is a potentially robust and is rich in reactive functional (-NH_2_,-OH, and –COOH) groups [23, 24].

However, the week mechanical strength of pure natural polymer hydrogels is major limitation in exploiting them in the controlled drug delivery. On the contrary, synthetic polymer hydrogels have strong mechanical strength. Moreover, synthetic polymers are easy to prepare and cheap. The properties of these polymers can be modified easily [25]. However, synthetic polymers have very low biodegradability and biocompatibility. Some synthetic polymers are hydrophobic in nature. The non-degradable solid wastes cause environmental pollution. Poly (acrylamide-co-acrylic acid) is a pH-sensitive, biocompatible, and hydrophilic synthetic polymer. The pH sensitivity and hydrophilicity of this polymer is due to the presence of carboxylic and amide groups.

Combination of natural and synthetic polymers has great significance because it improves the physical, chemical and biological properties and balances the mechanical properties which make them appropriate for complex biological system [26]. Furthermore, biodegradable and pH sensitive nature of N-succinyl chitosan and excellent mechanical strength of Poly (acrylamide-co-acrylic acid) can make this newly synthesized hydrogel highly suitable for controlled drug delivery system. According to the best of our knowledge, N-succinyl chitosan in combination with Poly (acrylamide-co-acrylic acid) is not reported in any form of formulation in the biomedical field. Our present work describes suitable and efficient new hydrogel formulation for drug delivery application. Indeed, the development of new hydrogel formulations with desirable properties is highly demanding in the current drug delivery systems.

Moreover, 5-Flouruoracil (5-FU) is an anti-tumor and anti-metabolite drug that has been used for few decades [27, 28]. It is usually used in the treatment of stomach cancer, gastrointestinal tract pancreas, colon, ovarian, breast, and head [29]. However, direct or intravenous administration of 5-FU has several side effects such as diarrhea, nausea, vomiting, soreness of the mouth, stomach pain, anemia, decrease in platelets, and white blood cells [30]. Cardiac, hematological, neural, and dermatological toxic side effects are also severe via intravenous administration. However, oral administration using controlled release formulations significantly reduced the side effects [31, 32]. Many polymer materials have been reported for the encapsulation and in vitro release of 5-FU [33]. In those studies, natural, synthetic as well as combination of both polymers were investigated. However, amount of 5-FU released from these carriers at acidic pH (stomach pH 1.2) was higher as compared to colon pH (pH 7.4) [34–37]. Ravichandran et al. synthesized pH-sensitive poly (N-vinyl pyrrolidone-acrylic acid)-polyethylene glycol hydrogels. These hydrogels released 62% 5-FU at pH 1.2. From 62% drug, 43% drug was released rapidly within 2 hours [35]. Shantha et al. synthesized pH-sensitive interpolymeric hydrogels of chitosan, N-vinyl pyrrolidone, and polyethylene glycol acrylate. These hydrogels were highly swellable in acidic medium (pH 1.2) as compared to pH 7.4. More than 60% of 5-FU was released at pH 1.2 in first two hours and 98% drug was released in 5 hours [36]. The pH-responsive hydrogels of chitosan-poly (acrylic acid) and chitosan-poly (vinyl alcohol) using gamma irradiation for the 5-FU delivery. More than 90% 5-Fu was released at pH 7.4 within 1 hour in case of chitosan-poly (acrylic acid), while higher 5-FU release occurred from chitosan-poly (vinyl alcohol) at acidic pH. However, drug was released in a short time and synthesis method involved gamma radiation which is very difficult to handle [37, 38]. Other pH-sensitive interpenetrating hydrogel networks of poly (N-acryloylglycine-chitosan) were reported for the 5-FU release. The 5-FU release results revealed higher release of drug at pH 2.1 [39]. However, our synthesized hydrogels showed lower drug release at pH 1.2 while higher drug release at pH 7.4 which is a significant advantage over reported formulations. The main objective of our work was to synthesize pH responsive semi-IPN of NSC/ Poly (AAm-co-AA) hydrogel for colon targeted rather than stomach targeted controlled 5-FU release. Therefore, pH-sensitive NSC was synthesized, followed by preparation and characterization of co-polymer hydrogel via FTIR, NMR, FESEM, XRD, and TGA. The equilibrium swelling ratio of the hydrogels in buffer solutions of pH 1.2 and pH 7.4 was investigated. 5-FU loading and encapsulation efficiency and in vitro release of 5-FU at pH 1.2 and pH 7.4 were observed. Moreover, the swelling behavior and drug release mechanism were studied using the kinetic models.

## Materials and methods

### Materials

Chitosan (medium molecular weight (M_w_ 190–310 kDa), degree of deacetylation 75–85%, viscosity = 200–800 cP), succinic anhydride, Poly (acrylamide-co-acrylic acid) (M_w_ 5,000,000), and 5-FU were obtained from Sigma Aldrich (Malaysia). Glutaraldehyde (25% solution) obtained from Unilab. Acetic acid, sodium hydroxide, methanol, and acetone were obtained from Friendemann Schmidt (Malaysia). Ethanol was purchased from John Koillin chemicals. D_2_O was obtained from Merck and sodium chloride, oxalic acid, potassium chloride, and potassium dihydrogen phosphate were purchased from Univar. These reagents were used without further purification.

### Methods

#### Synthesis of N-succinyl chitosan

N-succinyl chitosan was synthesized with slight modification following the already reported method [40]. In this method, 0.3 g chitosan was suspended in 50 ml aqueous solution of 5% acetic acid. This mixture was placed at hot plate at 50°C and stirred gently for 30 minutes. After that, dilution of the solution was done by adding 50 ml methanol. Furthermore, 1.5 g succinic anhydride was dissolved in 40 ml acetone and added into the chitosan solution drop wise under stirring for the succinylation of chitosan. The stirring was continued at 1400 rpm at 50°C for 24 hours. After that, mixture was further diluted with excess 1M NaOH solution to raise the pH of reaction mixture and in order to obtain clear solution. The stirring of this clear solution was continued for further 3 hours at the same temperature. Afterward, ethanol was added to form precipitates followed by filtration to separate the precipitates. These precipitates were dispersed in ethanol for 24 hours. Then, dispersed precipitated product was washed with acetone and ethanol to remove impurities and unreacted reactants and dried using freeze-dryer in vacuum oven for 3 hours at 50°C.

#### Degree of substitution (DS)

Degree of substitution of N-succinyl chitosan was found following the reported procedure [41]. In this method, aqueous solution of N-succinyl chitosan was prepared by adding 0.1 g of it in 20 ml distilled water and HCl was added dropwise to adjust the pH of solution to 2.0. After that, this acidic solution was titrated against 0.1 M NaOH. The degree of substitution was determined using the following Eq 1:

$$\text{DS }=\;\frac{177\;\times \text{ A}}{{\text{m}}_{\text{NSC}}-\;101\;\times \text{ A}}$$ (1)

$$\text{A }={\text{ V}}_{\text{NaOH}}\;\times {\text{ C}}_{\text{NaOH}}$$

Where, C_NaOH_ and V_NaOH_ are concentration and volume of NaOH, respectively. m_NSC_ is the mass of N-succinyl chitosan. 177 and 101 are molecular weights of glucosamine and succinyl group, respectively.

#### Synthesis of N-succinyl chitosan/ Poly (acrylamide-co-acrylic acid) hydrogels

Various amounts of N-succinyl chitosan and Poly (AAm-co-AA) were dissolved in distilled water. These reactants were mixed with specific amount of glutaraldehyde in the presence of 0.01M HCl solution to crosslink N-succinyl chitosan. Similarly, numerous hydrogels were synthesized using different amounts of N-succinyl chitosan and Poly (AAm-co-AA) with different crosslinking agent concentration. The mixtures were stirred under 60°C for 3–6 hours by magnetic stirrer. Then, the synthesized hydrogels were dried in vacuum oven at 60°C for 8 hours followed by physical properties characterization. The composition of synthesized hydrogels is given in the Table 1:

**Table 1:** Composition of hydrogels.

Hydrogel	NSC (mg)	Poly (AAm-co-AA) (mg)	GA(mg)
**SP1**	10	5	1
**SP2**	10	10	1
**SP3**	5	10	1
**SP4**	10	5	2
**SP5**	10	10	2
**SP6**	5	10	2
**SP7**	10	5	3
**SP8**	10	10	3
**SP9**	5	10	3

### Characterization of semi-interpenetrating network hydrogels

The synthesis of N-succinyl chitosan and hydrogels was analyzed through characterization using FTIR, NMR, powder XRD, TGA, and FESEM. Furthermore, SP2 hydrogel was characterized using FTIR, XRD and TGA. SP2 hydrogel shows good results among all the hydrogels in terms of swelling ratio, drug encapsulation and drug release. It is optimum and contains equal amount of NSC and Poly (AAm-co-AA).

#### Fourier transform infrared analysis

FTIR analysis of chitosan, NSC, Poly (AAm-co-AA) and SP2 hydrogel was performed using FTIR-ATR (Perkin Elmer Spectrum 400) spectrometer in transmittance mode from 4000 cm^-1^ to 500 cm^-1^ at resolution of 12 cm at 25°C.

#### Nuclear magnetic resonance (NMR) analysis

^1^H and ^13^C NMR spectra of chitosan and NSC were traced by NMR Bruker 600 MHz spectrometer at room temperature. The chitosan was dissolved in D_2_O and acetic acid while N-succinyl chitosan was dissolved in D_2_O.

#### X-ray diffraction (XRD)

The powder X-ray diffraction analysis of chitosan, NSC and SP2 hydrogel was performed at room temperature using Empyrean diffractometer. The scan rate was set at 1° min^-1^ at 2θ and accelerating voltage of 40 kV.

#### Thermogravimetric analysis (TGA)

TGA of NSC and SP2 hydrogel was carried out on a TGA Q 500 under nitrogen atmosphere. 3–5 mg of each sample was taken and scanned from 25°C to 700°C at a scan rate of 10°C/ min.

#### Morphology studies

Morphology of the freeze dried SP1, SP2, SP3 and SP4 hydrogels was examined using field emission scanning electron microscope (FESEM), Quanta FEG 450 at an accelerating voltage of 5kV. The freeze dried samples were coated with gold to avoid charging effects prior analysis.

### Equilibrium swelling ratio (ESR)

Swelling ratios of hydrogels were observed in physiological mediums of pH 7.4 and pH 1.2 at 37°C. Specific amount of each hydrogel was immersed in buffer solutions. At specific time interval, hydrogels were taken out, blotted with filter paper and weighted. The same procedure was repeated three times for all hydrogels and the average value was adapted to measure the equilibrium swelling ratio as stated in Eq 2.

$$\text{ESR }=\;\frac{{\text{W}}_{\text{f}}-{\text{W}}_{\text{i}}}{{\text{W}}_{\text{i}}}$$ (2)

Where, W_i_ is the initial weight of hydrogel and W_f_ is the final weight of the hydrogel.

#### Swelling kinetics

The swelling kinetics of the results obtained from swelling studies was analyzed by fitting the swelling data into non-linear second order rate Eqs 3,4 & 5 [42].

$$\frac{\text{t}}{{\text{SR}}_{\text{t}}}\;=\;\frac{1}{{\text{m}}_{\text{e}}}\text{t}+\;\frac{1}{{\text{m}}_{\text{e}}^{2}{\text{k}}_{\text{s}2}}$$ (3)

$${\text{SR}}_{\text{t }=}\;\frac{{\text{m}}_{\text{e}}^{2\;}k_{s2}\text{t}}{1+{\text{ m}}_{\text{e}}{\text{k}}_{\text{s}2}\text{t}}=\;\frac{{\text{r}}_{\text{o}}\text{t}}{1+{\text{m}}_{\text{e}}{\text{k}}_{\text{s}2}\text{t}}$$ (4)

$${\text{r}}_{\text{o }={\text{ m}}_{\text{e }}^{2}{\text{k}}_{\text{s}2}}$$ (5)

SR_t_is swelling ratio at time t, m_e_ is mass of hydrogel at swelling equilibrium, r_o_ is the initial rate of swelling, and k_s2_is rate constant.

#### Diffusion kinetics

The diffusion of water into the hydrogels can be examined through diffusion exponent, n and diffusion constant, k_D_. The n and k_D_ can be determined from fractional water uptake (F) as in Eq 6 [43].

$$\text{F}=\;\frac{{\text{m}}_{\text{t}}}{{\text{m}}_{\text{e}}}\;={\text{ k}}_{\text{D}}{\text{t}}^{\text{n}}$$ (6)

Diffusion coefficient (D) of water was also obtained by using Eq 7 [43].

$$\text{D }={\;\pi \text{r}}^{2}{\left(\frac{{\text{k}}_{\text{D}}}{4}\right)}^{\frac{1}{\text{n}}}$$ (7)

Where, r is the radius of cylindrical hydrogels.

### Drug loading and encapsulation efficiency

The drug loading and encapsulation of 5-FU into the hydrogels were studied using reported method [44, 45]. For this, 5-FU solutions were prepared in double distilled water and weighed amount of various hydrogels were immersed in drug solutions. Drug loaded wet hydrogels were carefully taken out from solution after 48 hours. The absorbance of free 5-FU in the supernatant was determined by UV-vis spectrometer at 266 nm. The concentration of free 5-FU was determined from the calibration curve (1–20 μg/ml). The drug loading and encapsulation efficiency were calculated using the following Eqs 8 & 9.

$$5-\text{FU loading efficiency }\left(\text{\%}\right)=\frac{\text{Total amount of }5-\text{FU in hydrogel}}{\text{Total amount of hydrogel}}\times \;100$$ (8)

$$5-\text{FU encapsulation efficiency }\left(\text{\%}\right)=\frac{\text{Actual loading of }5-\text{FU in hydrogel}}{\text{Theoretical loading of }5-\text{FU in hydrogel}}\;\times 100$$ (9)

### In vitro drug release

In vitro release of 5-FU was observed at 37°C in simulated gastric fluid (SGF, pH 1.2) and simulated intestinal fluid (SIF, pH 7.4). At specific time intervals, 3 ml of sample solution containing released drug was taken. The absorbance of released drug was determined by UV-vis spectrometer at 266 nm. Drug release experiments were observed in triplicate, average of drug release was calculated as in Eq 10.

$$5-\text{FU release }\left(\text{\%}\right)=\;\frac{\text{Released drug from hydrogel}}{\text{Total drug in the hydrogel}}\;\times 100$$ (10)

## Results and discussion

**Fig 1:**
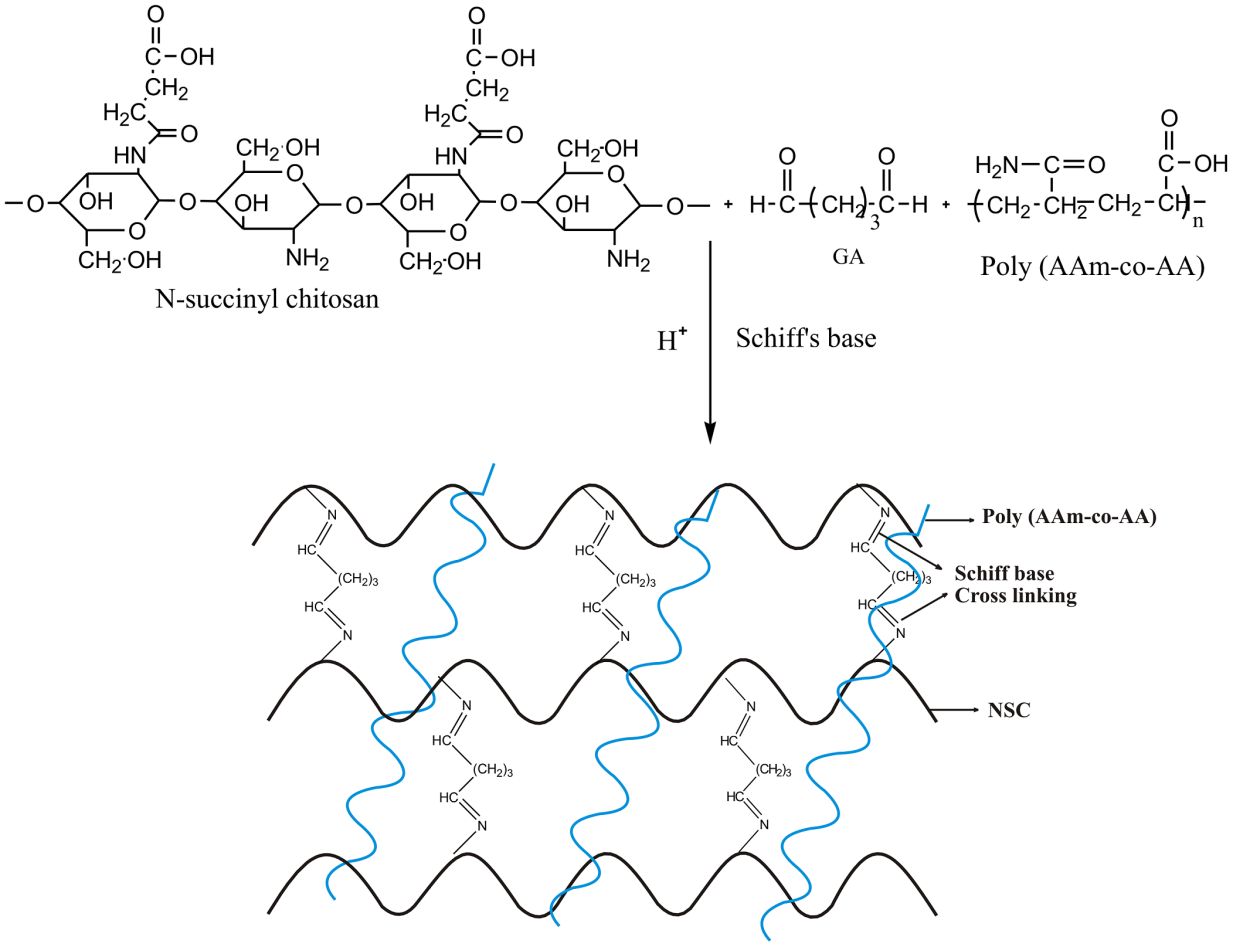
Proposed reaction mechanism of Schiff base crosslinking of NSC and formation of semi-IPN through embedding of Poly (AAm-co-AA) in NSC network.

N-succinyl chitosan was synthesized successfully. N-succinylation means the substitution of hydrogen from amino group by succinyl group. The degree of substitution means the extent of replacement of average number of one group by another specific group. In this study, N-succinyl chitosan was prepared by replacing the hydrogen of amino group of chitosan by succinyl group. The titration results revealed the degree of substitution was 0.51. Besides, NSC/ Poly (AAm-co-AA) hydrogels were synthesized. The proposed reaction mechanism is shown in Fig 1. This mechanism shows that amino groups of N-succinyl chitosan crosslinked through glutaraldehyde. Amino groups reacts with the aldehyde group to form N = C, known as Schiff base. This mechanism is known as Schiff base mechanism due to formation of Schiff base.

### Characterization of the hydrogels

#### Fourier transform infrared analysis

**Fig 2:**
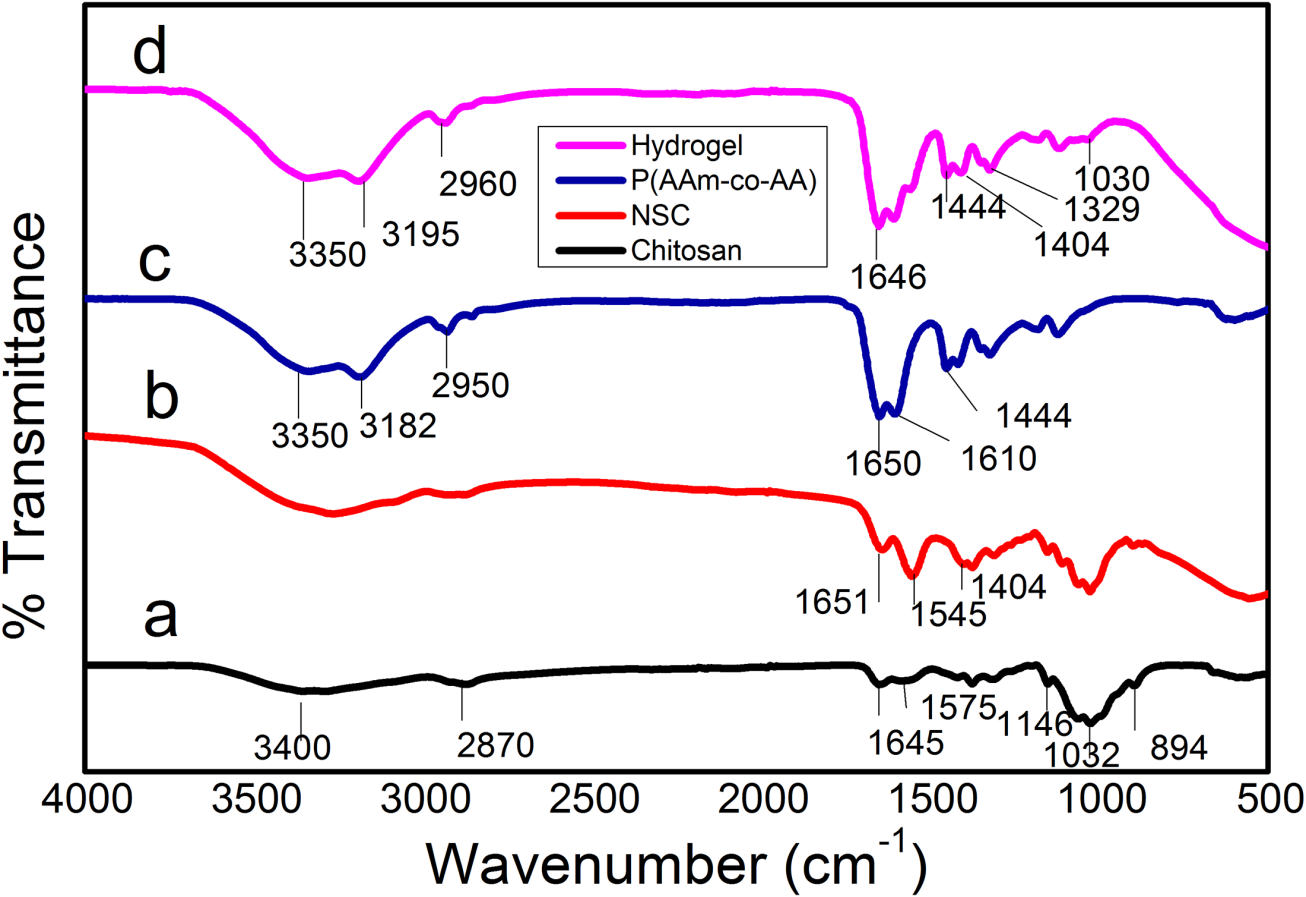
FTIR spectra of a) chitosan, b) NSC, c) Poly (AAm-co-AA), and d) SP2 hydrogel.

The FTIR spectra of chitosan, Poly (AAm-co-AA), NSC, and NSC/ Poly (AAm-co-AA) are shown in (Fig 2a, 2b, 2c & 2d). The characteristic peaks of chitosan were found at 3400 cm^-1^ (O-H stretching), 2870 cm^-1^ (C-H stretching vibration), 1645 cm^-1^ (primary amide), 1575 cm^-1^ (NH_2_ bending vibration), 1146 cm^-1^ (C-O-C stretching), 1032 cm^-1^ (C-O stretching), 894 cm^-1^ (pyranoid ring stretching). The band of NSC at 1404 cm^-1^ is due to asymmetric stretching of –COO^-^ ions. This absorption band provides a direct indication of NSC formation. In addition, the absorption peak at 1575 cm^-1^ vanished and one new peak emerged at 1545 cm^-1^ (secondary amine) further confirmed the formation of NSC. The spectrum of Poly (AAm-co-AA) has two significant peaks at 1650 cm^-1^ and 1444 cm^-1^ which represents amide group of acrylamide and symmetric stretching of -COO^-^ ions of acrylic acid. The spectrum of hydrogel showed some new peaks at 3350 cm^-1^ (O-H stretching), 3195 cm^-1^ (-NH_2_), 2960 cm^-1^ (C-H stretching), 1646 cm^-1^ (-C = N-), 1444 cm^-1^ (symmetric stretching of -COO^-^), 1404 cm^-1^ (asymmetric stretching of -COO^-^), and 1329 cm^-1^ (imide). The peaks at 1646 cm^-1^ and 1329 cm^-1^ provides strong evidence of formation of Schiff base between remaining amino groups of NSC. The presence of carboxylate ions and amide group in the semi-IPN hydrogels confirm the Schiff base and embedding of Poly (AAm-co-AA) within the hydrogel.

#### ^1^H and ^13^C Nuclear magnetic resonance spectra of chitosan and NSC

**Fig 3:**
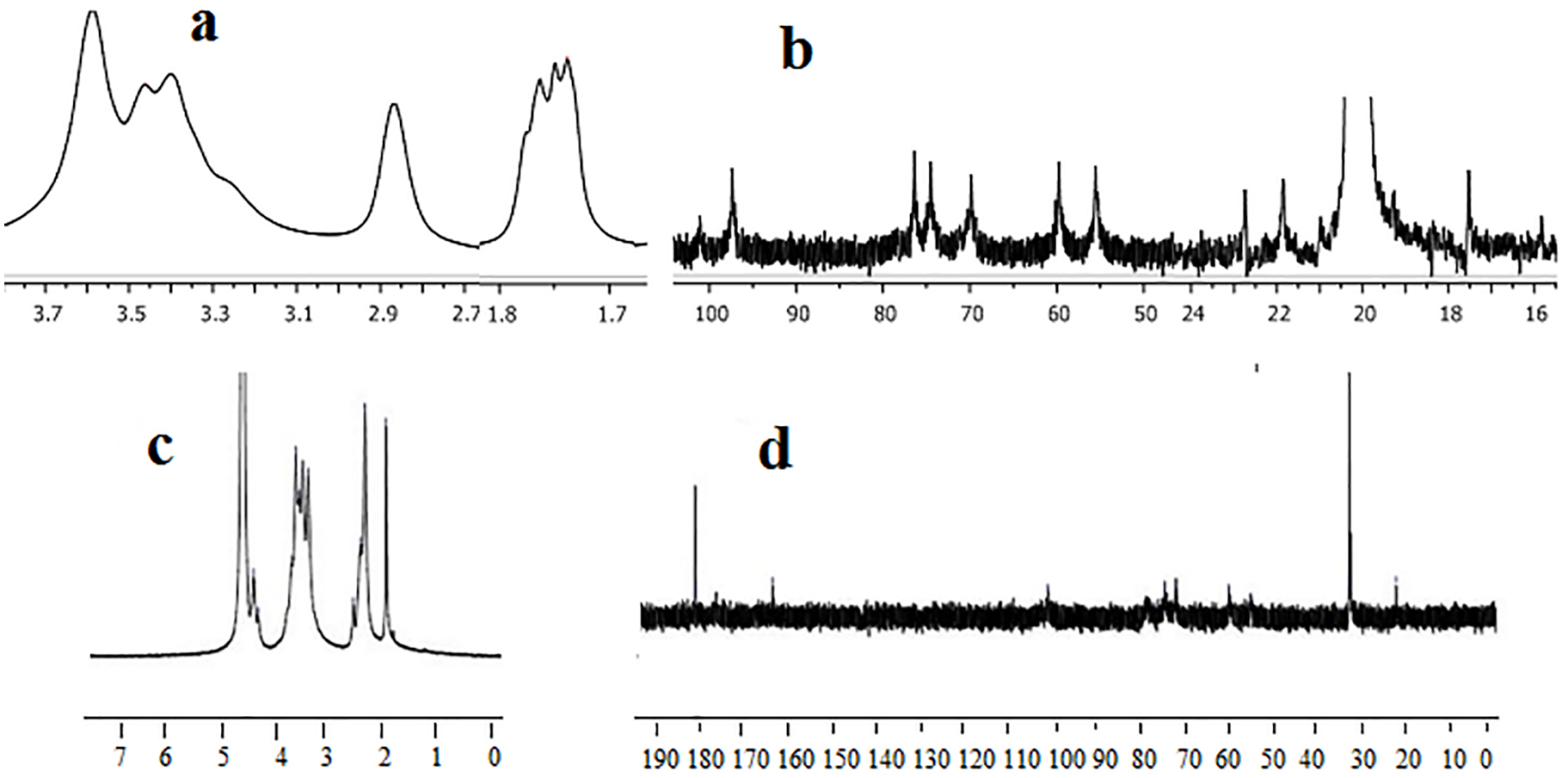
The NMR spectra of, a) ^1^H NMR of chitosan, b) ^13^C NMR of chitosan, c) ^1^H NMR of NSC, d) ^13^C NMR of NSC.

**(**Fig 3a, 3b, 3c & 3d) shows the NMR spectra of chitosan and NSC. Fig 3(a) represents the ^1^H NMR of chitosan. In this spectrum, the signals between 1.7–1.8 ppm show the presence of methyl hydrogen of acetamido groups. The signal at 2.9 ppm corresponds to H-2 of glucosamine unit. Moreover, the signals between 3.3–3.8 ppm confirm the presence of hydrogen bonded to C-3, C-4, C-5, and C-6 [46]. Furthermore, ^13^C NMR spectrum of chitosan shows that the peak at 20.13 ppm corresponds to -(CO)-CH_3_. The signals at 55. 56, 59.78, 70.0, 74.55, 76.30, and 97.40 ppm confirm C-2, C-6, C-3, C-4, C-5 and C-1 of chitosan main chain, respectively. The ^1^H and ^13^C NMR spectra of NSC are shown in (Fig 3c & 3d). In Fig 3(c), the peaks at 1.97, 2.37, and 2.46 ppm correspond to –NH-(CO)-CH_3_, –NH-(CO)-CH_2_- and –CH_2_-COO, respectively. The peak at 2.60 ppm corresponds to H-2 of glucosamine. The peaks from 3.45 to 3.77 ppm correspond to chitosan backbone hydrogen. H-1 of glucosamine unit is located at 4.4 ppm. The ^1^H NMR confirms that the site for formation of NSC is at –NH_2_. In Fig 3(d), ^13^C NMR spectrum of NSC shows that the peak at 22.12 ppm represents –NH-(CO)-CH_3_ while the large peaks at 32.41 and 32.69 ppm correspond to –CH_2_-CH_2_-. The small peaks at 54.83, 60.05, 71.97, 74.59, 76.14, and 101.16 ppm correspond to C-2, C-6, C-3, C-4, C-5 and C-1 of chitosan main chain respectively. In addition, the peaks at 174 and 177.34 ppm confirm the presence of –NH (CO)-CH_2_-CH_2_ and –COONa, respectively. These NMR spectral characteristics are in agreement with the literature [47, 48].

#### X-ray diffraction (XRD) analysis

**Fig 4:**
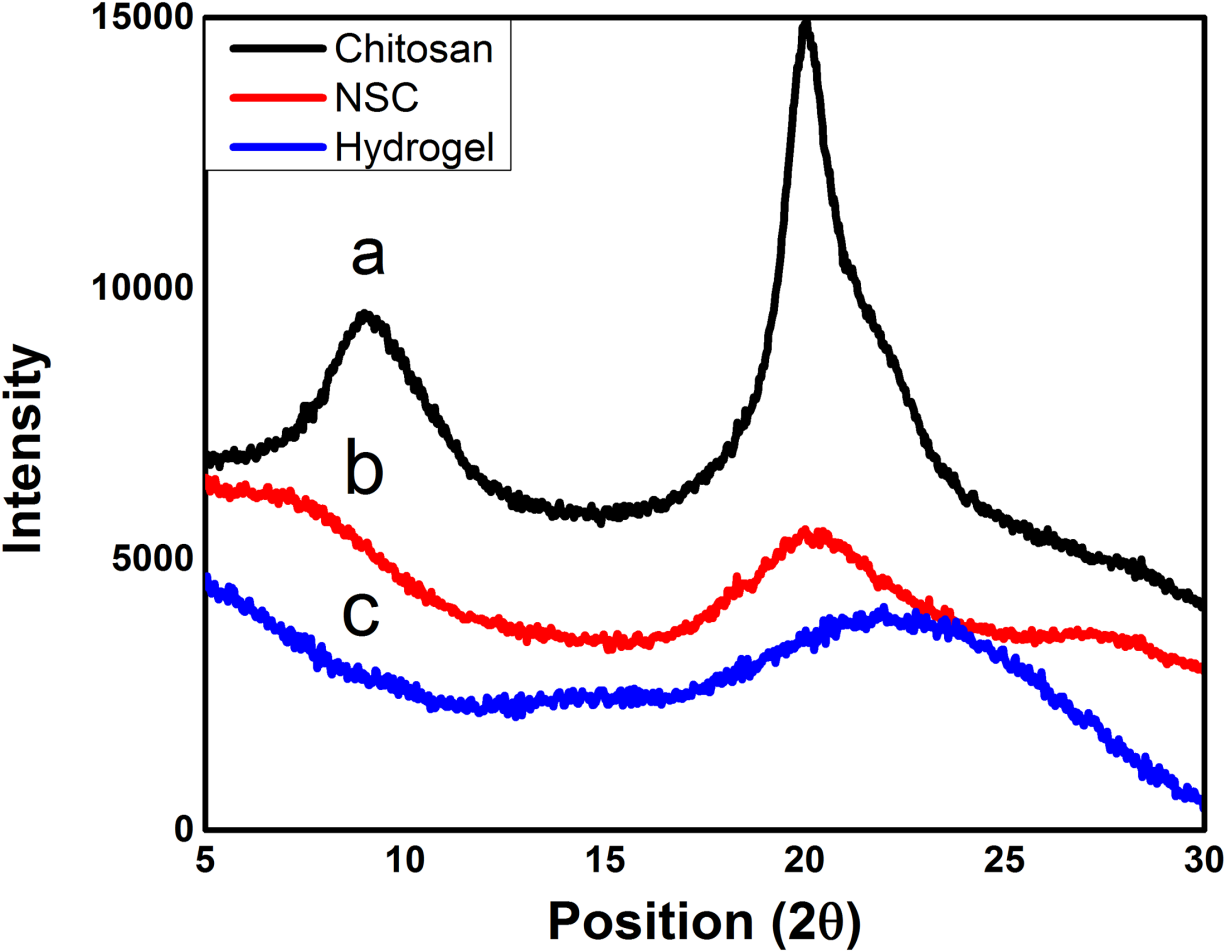
XRD spectra of the a) chitosan, b) NSC, and c) SP2 hydrogel.

XRD spectra of chitosan, NSC, and hydrogel are shown in (Fig 4a, 4b & 4c). According to these results, chitosan is semi-crystalline polysaccharide and shows two characteristic peaks, one strong peak at 2θ = 20° and other weak peak at 2θ = 10° [49]. This is owing to orderly arranged saccharide backbone and inter and intramolecular H-bonding among the functional groups present on the saccharide units which makes chitosan water insoluble and crystalline. The peaks at 2θ = 10° and 2θ = 20° are assigned to crystalline form I and II, respectively. It might be owing to the formation of intermolecular and intramolecular hydrogen bonds between hydroxyl and amino groups of chitosan. Furthermore, there is a regularity in the structure of chitosan molecules which makes it crystalline [50]. However, for NSC, the peak at 10° vanishes and the decrease in reflection of peak at 20° indicates succinylation of chitosan. This decrease in the intensity might be owing to the destruction of chitosan structure and reduction in the ability to form hydrogen bonds between hydroxyl and amino groups after successful modification [51]. Formation of NSC from chitosan also results in crystallinity destruction of chitosan. This change in XRD pattern also explains the predominant amorphous nature of NSC. Synthesis of hydrogel from NSC and Poly (AAm-co-AA) further decreased the reflection of peak at 20°. The marginal shifting of peak at 20° to 21.68° strongly indicates the breakage of intermolecular hydrogen bonds between hydroxyl and amino groups of the parent chitosan. All these findings evidently verified that the polymerization process destroyed the ordered structure of NSC and hydrogels [52]. This reduction in crystallinity would play a crucial role on influencing hydrogel degradability, water absorption and swelling ratio, as it is endorsed by results of the swelling ratio [53].

#### Thermogravimetric analysis (TGA)

**Fig 5:**
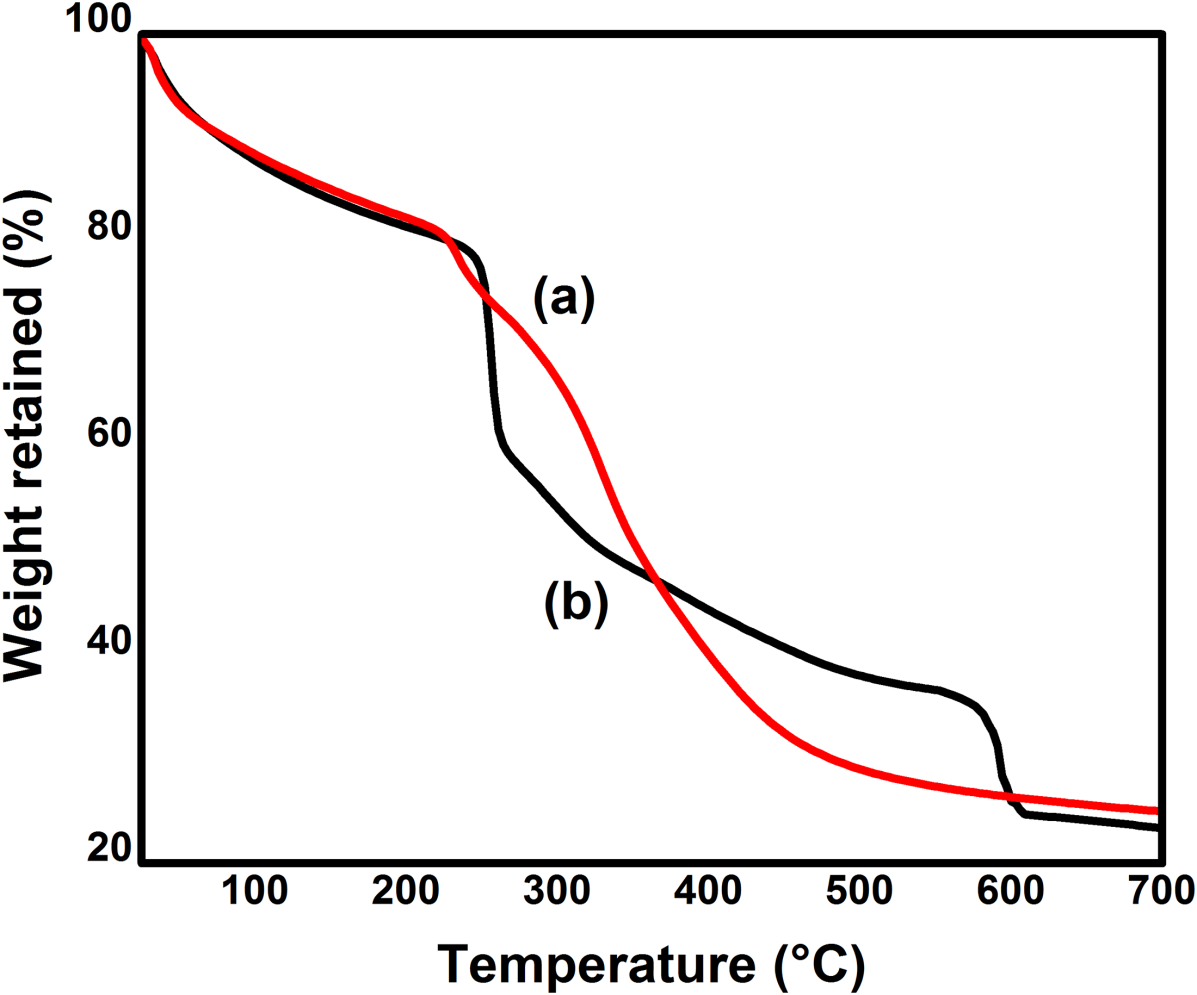
TGA graphs of a) NSC and b) SP2 hydrogel.

Thermal stability is very important for polymeric materials. For some thermally unstable biomaterials, thermal treatment during the process of manufacturing and long term usage at 37°C can lead to degradation of their mechanical properties and alteration of their cytotoxicity and biocompatibility. Thermogravimetric analysis is used to observe the thermal stability of the materials. The thermal properties of modified chitosan and hydrogels were observed. The reported literature reveals that chitosan usually shows two stage weight losses in the thermogram. Initially, chitosan loses its weight in the range of 50–100°C which is due to the presence of moisture, while second stage shows chitosan degradation in the range of 250–300°C [54]. In this study, chitosan was modified by succinic anhydride. TGA thermograms of NSC and hydrogels are shown in **(**Fig 5a & 5b). Modified chitosan loses its weight in three heating stages. Initially, 11.5% moisture weight loss can be observed in the range of 40–90°C. Modified chitosan showed 18% weight loss in the range of 200–300°C and this loss is due to degradation of polysaccharide backbone. A new stage of weight loss was observed in the range of 300–430°C due to degradation of succinyl group. At this stage, 30% loss in weight was observed [55]. This third stage of weight loss confirmed the successful succinylation of chitosan, and formation of NSC. Moreover, these three peaks appeared in the hydrogel curve along with one new significant peak at 530°C which showed the degradation of semi-IPN hydrogel. The weight loss occurred is 20% at this stage. This peak shows the successful synthesis of hydrogel. The resultant semi-IPN of NSC and Poly (AAm-co-AA) made the hydrogels less crystalline and thermally stable. The thermal stability mainly comes from the occurrence of crosslinking. Once the crosslinking occurs, polymers form hydrogels. This crosslinking of NSC was also responsible for the higher absorption of water or physiological medium which ultimately caused the increase in swelling properties of the hydrogels [56]. Furthermore, successful embedding of Poly (AAm-co-AA) contents resulted in an increase in the hydrophilic sites between the two neighboring crosslinked NSC macromolecules in the hydrogels which provided sufficient space for the water molecules to diffuse into the hydrogel. The larger the diffusion, higher will be the swelling properties of the hydrogels. Since the proposed hydrogel system is intended to be used for the release of drug from the varying medium or as a booster for use as a biomaterial. Furthermore, the release process is normally carried out at physiological temperature of 37°C, the polymer is totally stable in the vicinity of room temperature which normally varies between 27°C and 38°C [57].

#### Morphology studies

**Fig 6:**
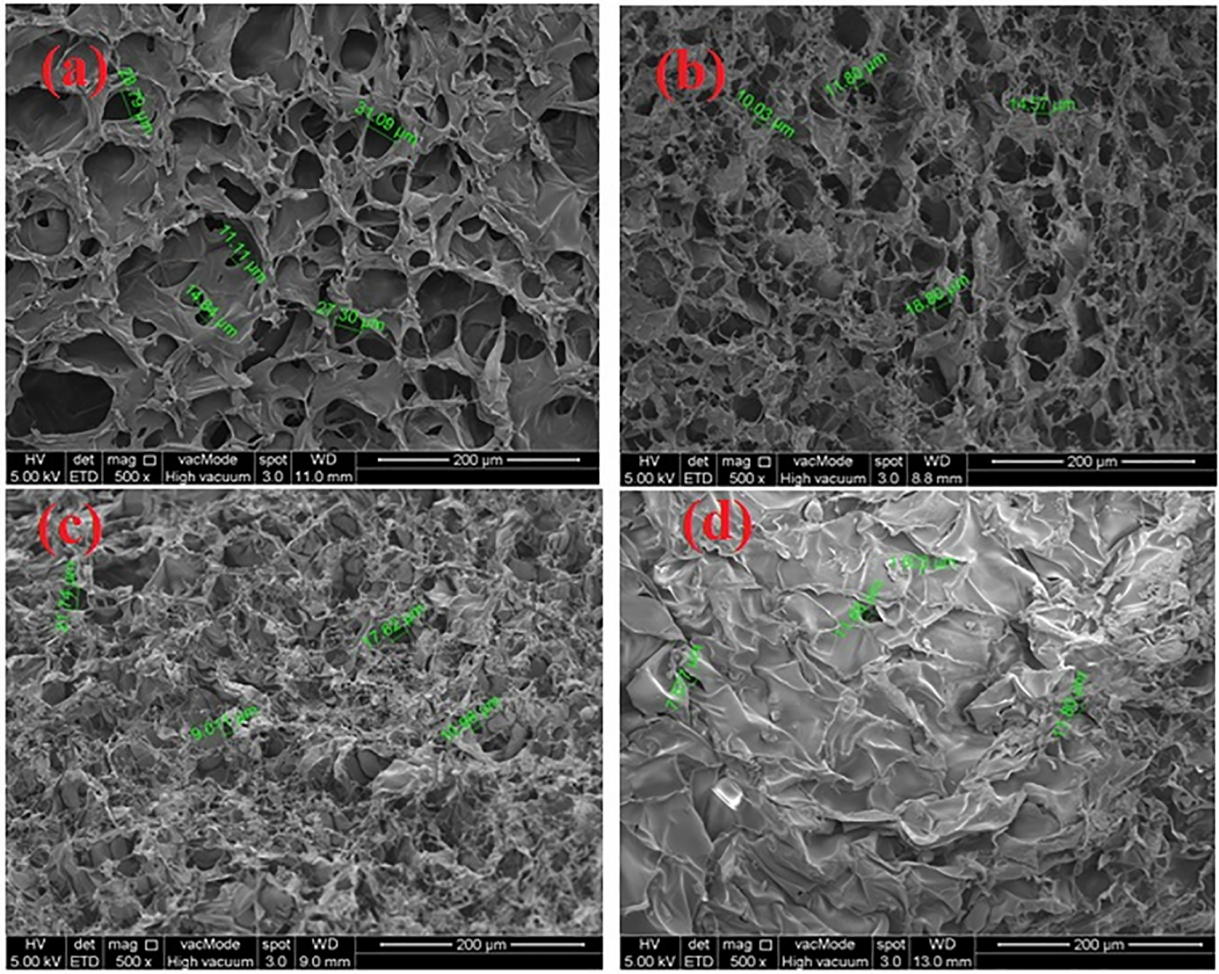
Represent the surface morphology of a) SP1, b) SP2, c) SP3, d) SP7.

The amount of water in a hydrogel, i.e. the volume fraction of water, and its free vs. bound water ‘character’ will determine the absorption (or partitioning) and diffusion of solutes through the hydrogel. Pores may be formed in hydrogels by phase separation during synthesis, or they may exist as smaller pores within the network. The average pore size, the pore size distribution, and the pore interconnections are important factors of a hydrogel matrix that are often difficult to quantitate. The effective diffusion path length across a hydrogel is estimated by the thickness of hydrogel. These factors, in turn, are most influenced by the composition and crosslinking density of the hydrogel polymer network. The porosity of hydrogels arose from evaporation of water during the process of freeze drying without affecting the morphology of hydrogels [58]. The porous construction of hydrogels enables them to swell, reduces flow resistance of water and enhances the rate to entrap drug. However, size and shape of drugs, its relative hydrophilic and hydrophobic character, and the availability of ‘free’ water molecules to hydrate and dissolve the drug molecules are important factors governing drug permeation through any particular hydrogel. The micrographs of freeze-dried hydrogels crosslinked with GA are shown in (Fig 6a, 6b, 6c & 6d). These micrographs show the high micro porosity, with a three dimensional interconnected microstructure by virtue of crosslinked network formation, resembling other reported macromolecular hydrogel structures. The interconnection between pores could be assigned to the crosslinked network formation in the hydrogels. The porosity of hydrogels also arose from evaporation of water during the process of freeze-drying, absorbed by the hydrophilic sites of the hydrogels. The porous construction of hydrogels enabled them to swell, reduces flow resistance of water and enhances the rate to entrap 5-FU due to hydroxyl, and carboxyl groups of NSC and carboxyl groups of acrylic acid. These groups form H-bonding with carbonyl group and -NH of the 5-FU. Hydrogels show different surface morphology due to change in the composition of the hydrogels. The change in the composition changed the network structure of the hydrogels. The Fig 6(a) shows the porous network structure of the hydrogel having pore size 11.11–31.09 μm. This porous network is owing to higher hydrophilicity of NSC because it is present in larger amount. Moreover, Fig 6(b) represents the surface morphology and pore size 10.03–18.80 μm of SP2 hydrogel in which Poly (AAm-co-AA) is embedded in large amount as compared to SP1. The embedding of this polymer resulted in apparently reduction in pore size [59]. However, the pore size is more uniform as compared to SP1 which shows the proper embedding of the Poly (AAm-co-AA). However, Fig 6(c) illustrates the dense network structure of the hydrogel. The pore size of this hydrogel was found to be in the range of 9–17 μm with less number of pores. This dense network might be due to embedment of larger amount of Poly (AAm-co-AA) and lower amount of NSC. Furthermore, pore walls of this hydrogels became thicker than those of SP1 and SP2. These thicker walls made the gel stronger [59]. Fig 6(d) demonstrates the surface morphology of SP7 which contained highest amount of crosslinking agent used in this study. The image shows the less porous structure having pore size ranging from 7–11 μm. The enhanced crosslinking agent decreased the number of pores and pore size which might be due to significant formation of tightly packed network structure. The increase in crosslinking agent resulted in reduction of hydrophilicity, swelling ratio, water diffusion, drug encapsulation and drug release.

### Equilibrium swelling study

**Fig 7:**
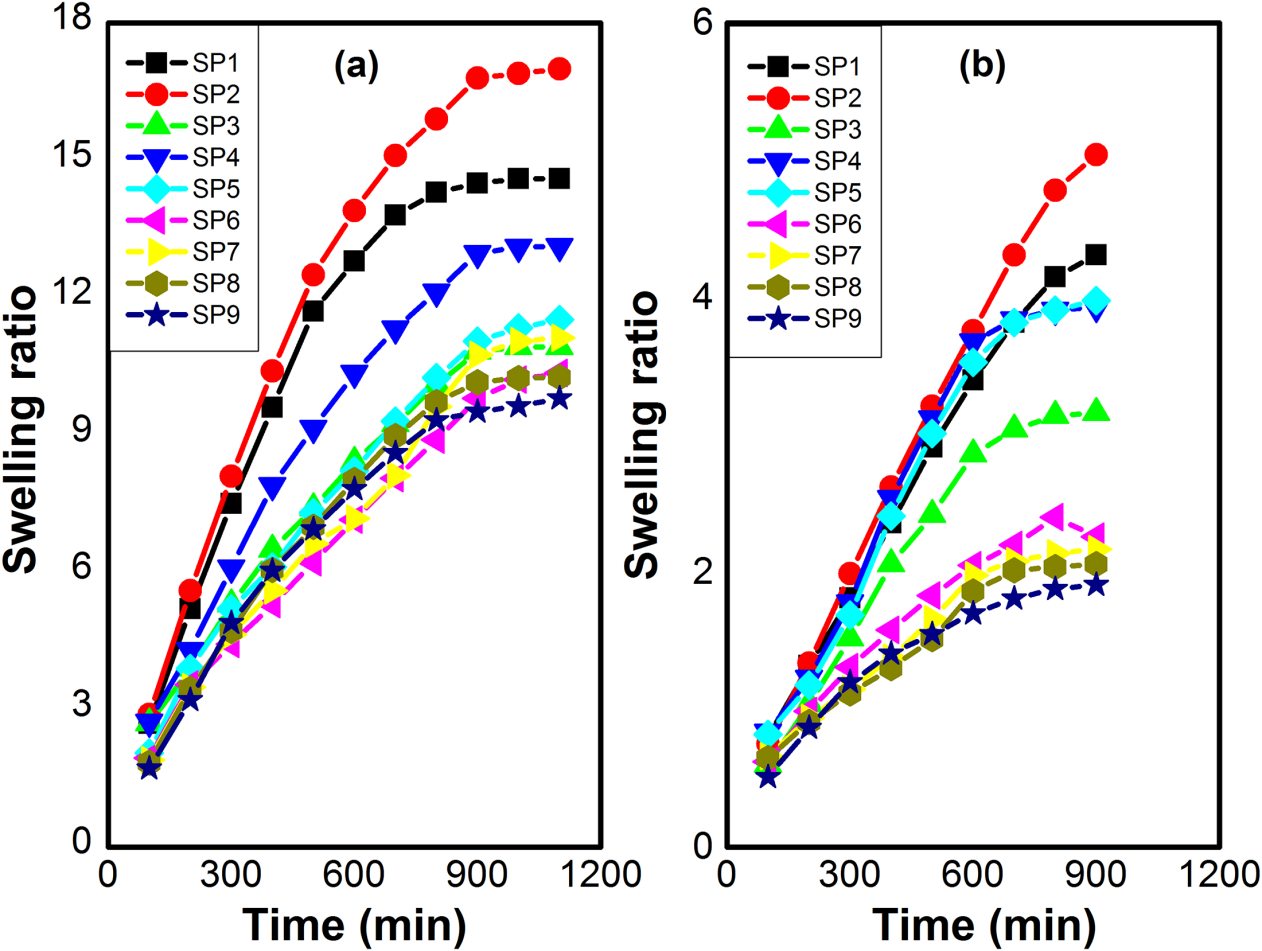
Swelling ratios of N-succinyl chitosan/ poly (AAm-co-AA) hydrogels at a) pH 7.4, b) pH 1.2.

Several hydrogels were synthesized using different concentrations of N-succinyl chitosan, Poly (AAm-co-AA) and crosslinking agent GA. These hydrogels were immersed in buffer solutions of pH 7.4 and pH 1.2. The swelling results demonstrated that swelling of hydrogel depended on the pH of swelling medium, amount of N-succinyl chitosan and Poly (AAm-co-AA), and crosslinking agent concentration [60]. The results of swelling ratio at pH 7.4 and pH 1.2 are shown in Fig 7. It can be seen that hydrogels showed higher swelling at pH 7.4 and lower swelling ratio at pH 1.2. The higher swelling at pH 7.4 might be due to presence of greater number of carboxylic groups. The carboxylic groups changed into carboxylate ions due to deprotonation. The carboxylate ions caused repulsion and swelling ratio increased [61]. However, swelling ratio of hydrogels at pH 1.2 might be due to protonation of amino groups and carboxylate ions present in the N-succinyl chitosan. These amino groups were less in number and swelling ratio was low. The composition of hydrogels had also influence on the swelling ratio. SP1, SP2, and SP3 hydrogels showed higher swelling due to less concentration of GA. Swelling ratio decreased with the increase in the concentration of GA. It can be observed from SP4, SP5, and SP6 hydrogels comprising higher concentration of GA. Furthermore, SP7, SP8, and SP9 showed lower swelling ratio due to higher concentration of GA as compared to the already described. Low concentration of GA resulted in higher swelling ratio due to looser network of hydrogels while increase in concentration of GA resulted in denser and tighter network of hydrogels [62] and hence lower the value of swelling ratio. The concentration of N-succinyl chitosan and Poly (AAm-co-AA) also influenced the swelling ratio of hydrogels. Hydrogels having higher concentration of N-succinyl chitosan had greater swelling ratio as compared to the hydrogels containing less concentration of swelling ratio. This is due to the high hydrophilicity of polysaccharide backbone and presence of hydrophilic groups such as –NH_2_,–COOH, and –OH groups. Furthermore, swelling ratio also affected with the change in concentration of Poly (AAm-co-AA). The swelling ratio increased with the increase in concentration of Poly (AAm-co-AA).

#### Swelling kinetics

Water permeation through hydrogel depends on the functional groups and the interaction between polymer and water [63]. The swelling results of hydrogels with different amounts of NSC, Poly (AAm-co-AA) and crosslinking agent obtained from the experimental observation were fitted in non-linear second order kinetic equation. The trend lines of non-linear fittings were obtained using excel and Origin Pro-8 Software. From these graphs, SR_t_ was calculated for each hydrogel using non-linear second order equation. The calculated swelling ratios are very close to the experimental swelling ratios. The regression coefficients (R^2^) of all these hydrogels are close to unity, while regressions show higher F values. The experimental and calculated swelling ratio, r_o_,k_s2_, and statistical parameters (R^2^ & F) are shown in Tables 2 and 3. For a good fitting of data to second order kinetic equation, R^2^ should be closer to unity and greater F value. Moreover, rate constant and initial rate of swelling should be comparable. The experimental and calculated ESR of each formulation is also very close to each other. From Tables 2 and 3, it can also be observed that with change in amount of NSC, Poly (AAm-co-AA) and crosslinking agent, there is a significant change in swelling parameters. It means these parameters are dependent on swelling of the gel and swelling depends on the crosslinking agent concentration and polymers amount. It might be owing to structural changes in the hydrogels and interactions between solvent and polymers. From the results, it can be clearly observed that all these values are according to literature reported. Hence, swelling data has significant fitting in second order rate equation [64].

**Table 2:** ESR at pH 1.2.

Hydrogels	K_s2_×10^−5^	r_o_×10^−3^	ESR_exp_	ESR_cal_	R^2^	F
**SP1**	7.7	8.2	4.43	4.6	0.97	545
**SP2**	1.7	7.6	5.04	5.1	0.97	1035
**SP3**	12.8	6.9	3.16	3.3	0.98	954
**SP4**	14.4	9.5	3.93	4.1	0.98	1342
**SP5**	11.6	8.9	3.98	4.1	0.97	1018
**SP6**	47.0	7.0	2.26	2.3	0.98	982
**SP7**	55.0	6.6	2.17	2.1	0.97	1234
**SP8**	64.3	6.5	2.06	2.0	0.98	1165
**SP9**	74.0	6.4	1.91	1.96	1.0	344

**Table 3:** ESR at pH 7.4.

Hydrogels	K_s2_×10^−5^	r_o_×10^−3^	ESR_exp_	ESR_cal_	R^2^	F
**SP1**	2.6	32.7	15.55	17.8	0.97	380
**SP2**	6.1	36.0	14.53	15.0	0.99	975
**SP3**	7.3	26.0	10.92	10.9	0.98	684
**SP4**	4.9	28.9	13.12	13.8	0.98	1210
**SP5**	4.8	22.8	11.65	12.0	0.98	1139
**SP6**	5.3	20.0	10.41	10.7	0.97	482
**SP7**	3.2	19.2	11.12	11.3	0.96	1090
**SP8**	5.0	21.1	10.26	10.9	0.98	1102
**SP9**	6.0	21.0	9.8	10.3	1.0	1254

#### Diffusion kinetics

The diffusion of water through hydrogel network is relevant to its drug release applications. The diffusion of water in the polymer is passive. The swelling medium or some external forces (polar or osmotic) can activate this passive diffusion. In order to understand diffusion kinetics, the fractional water uptake (F) of various synthesized hydrogels was determined from Eq 7. The water diffusion through the hydrogels can be assessed in terms of diffusion exponent (n), diffusion constant (K_D_), and diffusion coefficient (D). In this study, diffusion kinetics was assessed in terms of diffusion characteristics. K_D_ expresses the gel structure and n represents transport of solvent through the gel. The swelling data was fitted in Eqs 7 and 8 to obtain the diffusion kinetics. Diffusion kinetic values i.ek_D_, n and D at pH 1.2 and pH 7.4 are shown in Table 4. With increase in crosslinking concentration, n values decreased. The swelling of hydrogels, drug loading and release rates depend upon diffusion coefficients. The n value indicates transport of solvent through the hydrogels. When n is less than 0.5, then solvent diffusion through hydrogel is diffusion controlled (Fickian process) and solvent penetration into the hydrogel is much faster than polymer chain relaxation. Conversely, when n is closer to unity, the polymer relaxation controls the solvent penetration rather than diffusion controlled. It can also be said that swelling is dependent on polymer chains relaxation (Case-II transport). However, when 0.5 <n> 0.89, non-Fickian anomalous transport involves and swelling phenomenon is regarded as coupled diffusion and polymer chains relaxation. In this study, it can be observed that n values are between 0.5–0.89 and is non-Fickian anomalous diffusion, i.e., in these cases, rate of chain relaxation due to extensive swelling and diffusion of the gels are comparable.

**Table 4:** Diffusion parameters at pH 1.2/ pH 7.4.

Hydrogels	K_D_×10^−3^	n	D×10^−3^	R^2^	F
**SP1**	5/7	0.78/0.74	5/7	0.99/0.99	1210/3430
**SP2**	2.4/10	0.89/0.67	2/11	0.99/0.99	672/3590
**SP3**	5/13	0.8/0.63	5/16	0.99/0.99	702/3342
**SP4**	5/10	0.78/0.66	6/12	0.97/0.99	1103/3454
**SP5**	4.9/8	0.79/0.7	5/8	0.97/0.99	1240/2893
**SP6**	17/2	0.61/0.89	21/2	0.99/0.99	835/2793
**SP7**	20/6	0.58/0.72	27/7	0.98/0.99	316/2840
**SP8**	23/8	0.56/0.7	31/9	0.98/0.99	702/3031
**SP9**	18/8	0.6/0.7	24/9	0.99/0.99	545/2740

### Drug loading and encapsulation efficiency

The UV-vis spectrophotometer was used to determine the amount of drug loading in the hydrogels and encapsulation efficiency was calculated from the loaded drug and total drug. The results of loaded drug and encapsulation efficiency are shown in Table 5. These were calculated by varying the amount of NSC and Poly (AAm-co-AA) in the gel. It was found that drug loading and encapsulation efficiency were changed slightly with change in the amount of NSC and Poly (AAm-co-AA). These were increased with the increase in amount of NSC and Poly (AAm-co-AA) in the hydrogels. The increase in amount of NSC and acrylic acid increased the drug loading and encapsulation efficiency due to the increased interaction of active sites of NSC and acrylic acid with the drug molecules. 5-FU being a pyrimidine analog and ability to interact with active cites of hydrogels via hydrogen bonding. Increased drug loading and encapsulation efficiency was also due to decrease in crosslinking amount, resulting in significant increase in swelling of hydrogels. Enhanced swelling of the hydrogels results in the exposure of greater surface area to drug molecules which caused enhanced penetration and entanglement of the drug with the polymers.

**Table 5:** Drug loading and encapsulation efficiency.

Hydrogels	DL (%)	EE%
**SP1**	17.8	58.16
**SP2**	22.2	72.45
**SP3**	20.0	65.21
**SP4**	9.33	30.40
**SP5**	8.70	28.30
**SP6**	6.70	21.85
**SP7**	4.70	15.30
**SP8**	4.90	15.90
**SP9**	3.30	10.70

### In vitro release of drug

Release of a drug from the hydrogels will be controlled by the pore volume fraction, the pore sizes and their interconnections, the size of the drug molecule, and the type and strength of interactions of the drug with the polymer chains that make up the hydrogel network. In turn, the key factors that control the pore volume fraction, pore sizes and their interconnections are the composition of the network polymer chains and the crosslinking density. The interactions of the drug molecules with the network chains will be determined by their respective compositions. Besides, the release of the drug also depends on the pH of the medium during the release process. Amount and release rate of the drugs vary according to the pH of the medium. The pH dependent drug release is due to the interaction of active sites of the hydrogels with the medium and absorption phenomenon. Furthermore, anticancer drug release can be triggered by taking advantage of the pH of the intratumoral environment or the intracellular environment. In nonmalignant body tissue, pH is maintained near 7.4. Whereas, pH of the cancerous tissues is 6.5–7.2 (slightly acidic to neutral) [65]. Literature reveals the slight acidic (around pH 6.5) microenvironment of the solid tumor site [66, 67]. Moreover, the pH of solid tumors varies in the range of approximately 5.7 to 7.6 due to accumulation of acidic metabolites that are the result of low blood pressure and hypoxia caused by abnormal tumor vessels [68].

**Fig 8:**
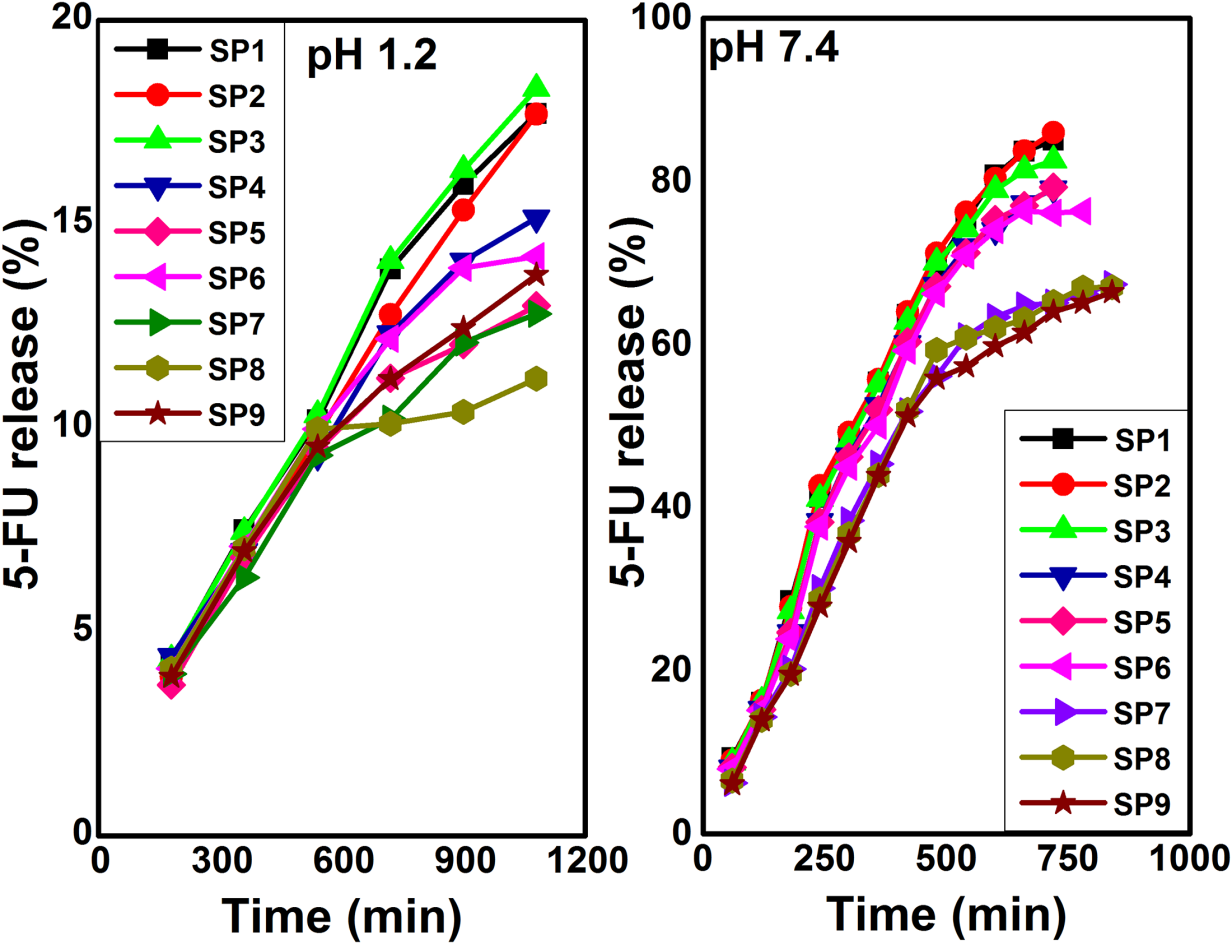
Drug release at pH 1.2 and pH 7.4.

In our work, 5-FU loaded hydrogels were evaluated through in vitro release in buffer solutions of pH 1.2 and pH 7.4 and the results are shown in Fig 8. The results of 5-FU release % were drawn against time. 5-FU release was observed to be dependent on NSC and Poly (AAm-co-AA) amount, crosslinking concentration, the nature and volume of physiological medium. The maximum release of encapsulated 5-FU was observed to be 15–20% at pH 1.2. This insignificant small release of drug at pH 1.2 is due to the protonation of carboxylate ions of NSC and acrylic acid. The protonation of carboxylate ions results in shrinkage of the NSC/ Poly (AAm-co-AA) hydrogels. This shrinkage of hydrogels causes low swelling. The higher amount of 5-FU released at pH 7.4 from hydrogel may be due to significant swelling of hydrogel. This remarkable drug release is owing to higher electrostatic repulsion of –COO^-^ ions of NSC and acrylic acid. The initial burst release from hydrogels was observed followed by sustained release. This burst release might show the presence of 5-FU on the surface of hydrogels and high concentration gradient between 5-FU and buffer solution [69]. Moreover, release profile followed the swelling trend of the hydrogels [70]. The increased concentration of NSC and Poly (AAm-co-AA) resulted in maximum release of drug. SP2, SP5, and SP8 contained same amount of NSC and Poly (AAm-co-AA) but different GA while SP1, SP4, and SP7 also possessed same amount of NSC and Poly (AAm-co-AA) but different GA. Similarly, SP3, SP6, and SP9 formulations contained same amount of NSC and Poly (AAm-co-AA) but different GA. The 5-FU release (%) in case of SP2, SP5, and SP8 formulations was 85.99%, 79.27%, and 67.02%, respectively. Furthermore, release (%) was 85.02%, 79.1%, and 67.37% in the case of SP1, SP4, and SP7 formulations. Similarly, SP3, SP6, and SP9 showed 82.56%, 76.32%, and 66.4% drug release. Hence, from the above mentioned results, it can be concluded that SP2, SP5, and SP8 showed higher release rates as compared to rest formulations which attributes to the existence of greater hydrophilic moieties and higher swelling nature. The crosslinking agent also affected the release rate of 5-FU. SP1, SP2, and SP3 were crosslinked with 1mg GA, SP4, SP5, and SP6 were crosslinked with 2mg GA while SP7, SP8, and SP9 were crosslinked with 3mg GA. The highest drug release (%) was observed from SP1, SP2, and SP3. If we further investigate the drug release profile of SP1, SP2, and SP3, it can be seen that 85.99% drug was released from SP2 which is highest among all the hydrogels. Furthermore, least drug release (%) was found from SP7, SP8, and SP9 formulations due to higher GA concentration. This was due to the fact that higher crosslinking concentration, free volume as well as swelling of hydrogels decreased significantly, thereby hindered the transport of drug molecules through the gel matrix into the release medium. Thus, NSC/ Poly (AAm-co-AA) gels found to be efficient in 5-FU release in a controlled manner at pH 7.4 while protected its release at pH 1.2, which is pH of stomach. The release profile of 5-FU fulfilled the basic requirement of US pharmacopeia (USP XXIV). According to this, minimum of 80% drug release at pH 7.4 is the standard for oral drug delivery [71]. If we compare the release profile of 5-FU from the already reported literature to our work, the results reveal the controlled and targeted release of 5-FU at pH 7.4 which is our goal to synthesize the hydrogels. All the reported hydrogels released the maximum drug at pH 1.2 within first 2 hours. In other words, we can say that 5-FU was released in a controlled fashion as compared to the reported literature which is a significant advantage of our work. These hydrogels were found to be efficient as hydrophilic drug carriers. The drug release was owing to easy flow of drug through the interconnected pores. Moreover, these hydrogels can be exploited for tissue engineering applications. Excellent interconnected porous network allows an efficient nutrient transfer and gaseous exchange, which can enhance the cell proliferation and cell survival on these hydrogel matrices.

#### Drug release kinetics

Drug release data of the hydrogels were fitted into the following Eqs 11, 12 and 13 to evaluate drug release kinetics. These equations are Donbrow-Samuelov, Higuchi, and Korsmeyer-Peppas model. Origin Pro 8 software was used to determine the release kinetics similar to swelling kinetics.

1. Donbrow-Samuelov model
  $${\text{m}}_{\text{Dt}}={\text{ m}}_{\text{De}}+{\text{ K}}_{0}\text{t}$$ (11)
2. Higuchi model
  $${\text{m}}_{\text{Dt}}={\text{ m}}_{\text{De}}+{\text{ K}}_{\text{H}}{\text{t}}^{1/2}$$ (12)
3. Korsmeyer-Peppas model
  $$\text{FD}=\;\frac{{\text{m}}_{\text{Dt}}}{{\text{m}}_{\text{De}}}\;={\text{ K}}_{\text{KP}}{\text{t}}^{\text{n}}$$ (13)

Where, m_Dt_ and m_De_ are the drug released at specific time and at equilibrium, respectively. K_o_, K_H_ and K_KP_ are constants of each model analogous to geometrical parameters of drug dosage and n is a diffusion exponent which explains drug release mechanism. All these models were applied to drug release data and found that m_De_ values for zero order and Higuchi model did not closely resemble the m_De_ of experimental value. The statistical parameter such as R^2^ was very close to unity, while F values were also very high for this release data. However, the drug release data showed close fitting to Korsmeyer-Peppas model. The n and K_KP_ values of different formulations were obtained from this model and are shown in Table 6. It is already described that n value suggests the drug release mechanism. If n < 0.45, drug release follows Fickian diffusion behavior, n < 0.89 elucidates anomalous transport (non-Fickian), and n ≥ 0.9 explain the drug release dependent on relaxation or swelling of system (Case-II transport). In this study, n values range from 0.49 to 0.84 when drug release occurred at pH 1.2. On the other hand, n values range from 0.90–0.95 when drug release occurred at pH 7.4. Hence, it is suggested that drug release followed non-Fickian diffusion controlled release of drug at pH 1.2 while Case-II transport of drug at pH 7.4 [72]. The drug release followed non-Fickian transport and was dependent on the crosslinking agent concentration. The n values are greater at low concentration of GA while smaller at high concentration of GA. The higher n values at lower GA concentration describes the loose gel network and greater swelling ratio. Unlike, high n values attribute to tight network of gel matrices at higher concentration of GA, thereby leading to least swelling. Similarly, K_KP_ values range between 0.002–0.027 for all formulations. The smaller values of K_KP_ indicate the lesser interactions between the gel matrices and drug. The statistical parameters such as R^2^, and F values highly indicated close fitting of drug release data to this model and strongly sports the accuracy of results. These values are also shown in Table 6. Hence, it can be concluded that, the drug release rate is dependent on the swelling and relaxation of polymer system.

**Table 6:** Drug release kinetics at pH 1.2/ pH 7.4.

Hydrogels	K_KP_ 1.2/7.4	n	R^2^	F
**SP1**	0.0036/0.006	0.79/0.92	0.99/0.99	1230/2302
**SP2**	0.0026/0.004	0.84/0.93	0.99/0.99	1090/2427
**SP3**	0.0034/0.005	0.80/0.94	0.99/0.99	1706/2346
**SP4**	0.0075/0.002	0.69/0.95	0.99/0.99	1675/2267
**SP5**	0.0093/0.002	0.69/0.92	0.99/0.99	1534/2163
**SP6**	0.0071/0.007	0.70/0.92	0.99/0.98	1589/2278
**SP7**	0.011/0.0028	0.62/0.90	0.99/0.99	1633/2304
**SP8**	0.027/0.0028	0.49/0.90	0.98/0.98	1677/2416
**SP9**	0.014/0.0027	0.59/0.90	0.99/0.99	1548/2356

## Conclusion

NSC was synthesized and characterized by FTIR, ^1^H, and ^13^C NMR. Several hydrogels were prepared by crosslinking of NSC using GA as a crosslinking agent. Poly (AAm-co-AA) was embedded during the synthesis of hydrogels through Schiff base formation between the amino groups of NSC. These hydrogels were prepared by varying amounts of NSC, Poly (AAm-co-AA), and GA. Hydrogels were characterized by FTIR, XRD, FESEM, and TGA. FTIR, XRD, and TGA data confirmed the successful synthesis of hydrogel while FESEM showed porous morphology of hydrogel. The swelling characteristics were highly dependent on amount of NSC, Poly (AAm-co-AA), GA concentration, and pH. Swelling kinetics and diffusion parameters were evaluated. The 5-FU loading and encapsulation efficiency were in the range of 3.3% to 22.2% and 10.7% to 72.5%, respectively. In vitro release of 5-FU was studied at pH 1.2 and pH 7.4 with different amounts of polymer and GA. The drug release studies showed the significant effect of pH, polymer amount and concentration of GA on the release profile. Hydrogels showed 5-FU release in the range of 64.0% to 85.99% at pH 7.4 while 13.33% to 19.64% at pH 1.2. Drug release kinetics and statistical parameters showed non-Fickian diffusion controlled release of 5-FU and closely fitted to Korsmeyer-Peppas model. From the results, it can be concluded that SP2 hydrogel showed significant drug loading and encapsulation efficiency and in vitro drug release properties which can be considered as optimum. It can also be concluded that this hydrogel is useful for oral delivery of hydrophilic drug. This hydrogel can be investigated as hydrophobic drug carrier. This hydrogel has high mechanical strength and can be explored for tissue engineering applications. Excellent interconnected porous network would allow an efficient nutrient transfer and gaseous exchange, which can enhance the cell proliferation and cell survival on these hydrogel matrices.
