# Correction: pH responsive N-succinyl chitosan/Poly (acrylamide-co-acrylic acid) hydrogels and *in vitro* release of 5-fluorouracil

**DOI:** 10.1371/journal.pone.0185505

**Published:** 2017-09-21

**Authors:** Shahid Bashir, Yin Yin Teo, Sumaira Naeem, S. Ramesh, K. Ramesh

The Funding statement is incorrect. The correct statement is: This work was supported by Ministry of Higher Education FRGS grant (FP012-2015A) and University of Malaya Prototype Research Grant Scheme (PR002-2015B).
